# Application of multi-echo susceptibility weighted imaging in the evaluation of brain capillary telangiectasias

**DOI:** 10.3389/fneur.2025.1593152

**Published:** 2025-11-03

**Authors:** Yun Meng, Kaixi Xu, Xinjian Chen, Xingru Xu, Baodong Gu, Yunlei Zhao, Xianjun Ma

**Affiliations:** Department of Radiology, Lianyungang Traditional Chinese Medicine Hospital Affiliated to Nanjing University of Chinese Medicine, Lianyungang, China

**Keywords:** brain capillary telangiectasias, magnetic resonance imaging, multi-echo susceptibility weighted imaging, quantitative susceptibility mapping, venous imaging

## Abstract

**Objective:**

To explore the diagnostic value of high-resolution 3.0 T multi-echo susceptibility weighted imaging (SWI) technique for brain capillary telangiectasias (BCT).

**Methods:**

In this study, 36 BCT patients’ conventional MR images and multi-echo SWI were retrospectively collected, and the BCT imaging features on conventional MR images, varied echo time SWI and derived quantitative susceptibility mapping (QSM) images, were reviewed and analyzed.

**Results:**

The patients were 25 males and 11 females, ranging in age from 28 to 90 years, with an average age of (64.75 ± 11.08) years. The positive rate of diffusion-weighted imaging (DWI) image for BCT was 36.9%, that of multi-echo SWI long TE sequence for BCT was 100%, and that of QSM map was 85%. The positive rate of QSM was better than that of DWI, and the difference was statistically significant ( $\chi^{2}$ = 334.609, *p* < 0.001). The long TE (22.5 ms) of multi-echo SWI sequence showed the central vein of BCT 72.4% (498/688), and the display rate of QSM map was 55.4% (324/585). The detection rate of central vein of BCT was different between the two sequences, and the multi-echo SWI sequence was better than the QSM map ( $\chi^{2}$ = 39.937, *p* < 0.001).

**Conclusion:**

Multi-echo SWI technique is the most sensitive sequence for BCT diagnosis and lesion display, and the central vein and peripheral drainage vein of BCT lesion are clearly displayed with the extension of TE time.

## Introduction

Brain capillary telangiectasias (BCT) are low-flow, occult vascular malformations consisting of a small cluster of dilated capillary-like vessels. In most cases, they are incidentally discovered and have no obvious clinical symptoms. They are difficult to detect on CT and conventional MRI, often being misdiagnosed as microhemorrhages, demyelination, or tumors (1, 2). Identifying BCT is crucial to avoid misdiagnosis and prevent unnecessary interventions.

With the widespread application of high-resolution 3.0 T MRI susceptibility-weighted imaging (SWI) and quantitative susceptibility mapping (QSM), the frequency of BCT detection has increased. SWI and QSM are sensitive to magnetic susceptibility differences between tissues, providing a non-invasive method for brain venous imaging. By detecting magnetic field changes around vessels caused by varying deoxyhemoglobin content in low-flow dilated capillaries, these techniques are highly sensitive to small venous structures, blood metabolites, iron deposition (3–5), brain iron content, blood oxygen saturation, and calcification (6, 7). QSM has been widely applied in clinical diagnosis and scientific research, such as the assessment of cerebral microbleeds (8, 9) or the evaluation of hematoma size (10–12), the research on neurodegenerative diseases related to iron metabolism (13, 14), the differentiation between hemorrhage and calcification (15, 16), and vascular malformations (17–19).

Recently, a new sequence has emerged that can provide multi-echo SWI images while simultaneously generating QSM images (20). However, this technology has not yet been applied to the diagnosis of BCT. This article collected 36 cases of BCT examined using high-resolution 3.0 T multi-echo SWI technology, aiming to explore the re-evaluation and diagnostic value of BCT.

## Materials and methods

### Patients

Retrospective collection of 1,705 patients who underwent SWI or QSM examination in Lianyungang Hospital of Traditional Chinese Medicine from January 1, 2023, to July 30, 2024, among which 385 patients were diagnosed with BCT, accounting for 22.58%. Multi-echo SWI examination was performed on 36 BCT patients. There were 25 males and 11 females, aged 28–90 years, with an average age of (64.75 ± 11.08) years. Clinical manifestations included asymptomatic in 6 cases, headache and dizziness in 13 cases, headache accompanied by blurred vision in 5 cases, speech impairment in 3 cases and limb weakness in 9 cases. Inclusion criteria: (1) Patients who were diagnosed BCT. (2) Underwent multi-echo SWI examination of the head; (3) Complete clinical data. Exclusion criteria: (1) Non-BCT patients. (2) Without multi-echo SWI examination. (3) Poor image quality.

### Methods

All patients were scanned using 3.0 T MRI scanner at our hospital (uMR790, United Imaging Healthcare), with a 32-channel phased-array head coil. Routine sequences included T1WI, T2WI, T2-FLAIR, DWI, ADC, and multi-echo SWI. Conventional MRI parameters were as follows: 3D T1WI (TR 7.1 ms, TE 2.9 ms, slice thickness 1 mm, matrix 256 × 240, FOV 24.0 cm × 25.6 cm, flip angle 8°, NEX 1, Duration 1 min 40 s); T2WI (TR 5,421 ms, TE 132.44 ms, matrix 448 × 390, FOV 20.0 cm × 23 cm, flip angle 8°, NEX 1, Duration 1 min 22 s); FLAIR (TR 8,000 ms, TE 130.72 ms, matrix 288 × 200, FOV 20.0 cm × 23 cm, flip angle 150°, NEX 1, Duration 1 min 36 s); DWI (TR 2,000 ms, TE 67 ms, *b*-values 0/1,000 s/mm^2^, matrix 153 × 160, FOV 22.0 cm × 23 cm, bandwidth 350 kHz, flip angle 90°, NEX 2, slice thickness 5 mm, Duration 1 min 23 s). Multi-echo SWI (slice thickness 0.8 mm with no gap, 152 slices, matrix 285 × 250, FOV 20.0 cm × 22.8 cm, bandwidth 350 kHz, flip angle 15°, NEX 1, TR 28.1 ms, and seven TEs of 3.3, 6.5, 9.7, 12.9, 16.1, 19.3, and 22.5 ms, Duration: 4 min 54 s).

### Image processing and image analysis

After the completion of the multi-echo SWI sequence scanning, the phase map, amplitude map, SWI map, MinP map, and QSM map were reconstructed inline. Based on the following criterion to diagnose the BCT: single or multiple round-like hypointense lesions are observed in the brain parenchyma, with punctate hyperintensity in the center on SWI amplitude images; or on QSM images, single or multiple round-like hyperintense lesions are seen in the brain parenchyma, with punctate hypointensity in the center, presenting the typical “target sign.” With or without multiple, increased, thickened, and tortuous veins around the lesions, which appear as strip-like hypointensity connected to the lesions. One radiologist with 15 years of experience (MY), one with 30 years of experience (XK), and one neurologist with 16 years of experience (XX) independently read the images, and in case of disagreement, they engaged in discussions to reach a consensus.

### Statistical analysis

Statistical analyses were performed using IBM SPSS Statistics Version 23.0 (IBM Corp., Armonk, NY, United States). For categorical variables, the detection of positive signs was reported as both absolute counts and percentages, with the positive rate calculated as the number of detected positive signs divided by the total number of positive signs. Fleiss’ Kappa was employed to assess the inter-reader agreement. Intergroup comparisons were conducted using the Pearson chi-square test or Fisher’s exact test (two-sided), with statistical significance set at *p* < 0.05 and high significance.

## Results

### Imaging findings of BCT lesions in multi-echo SWI and QSM

**Figure 1:**
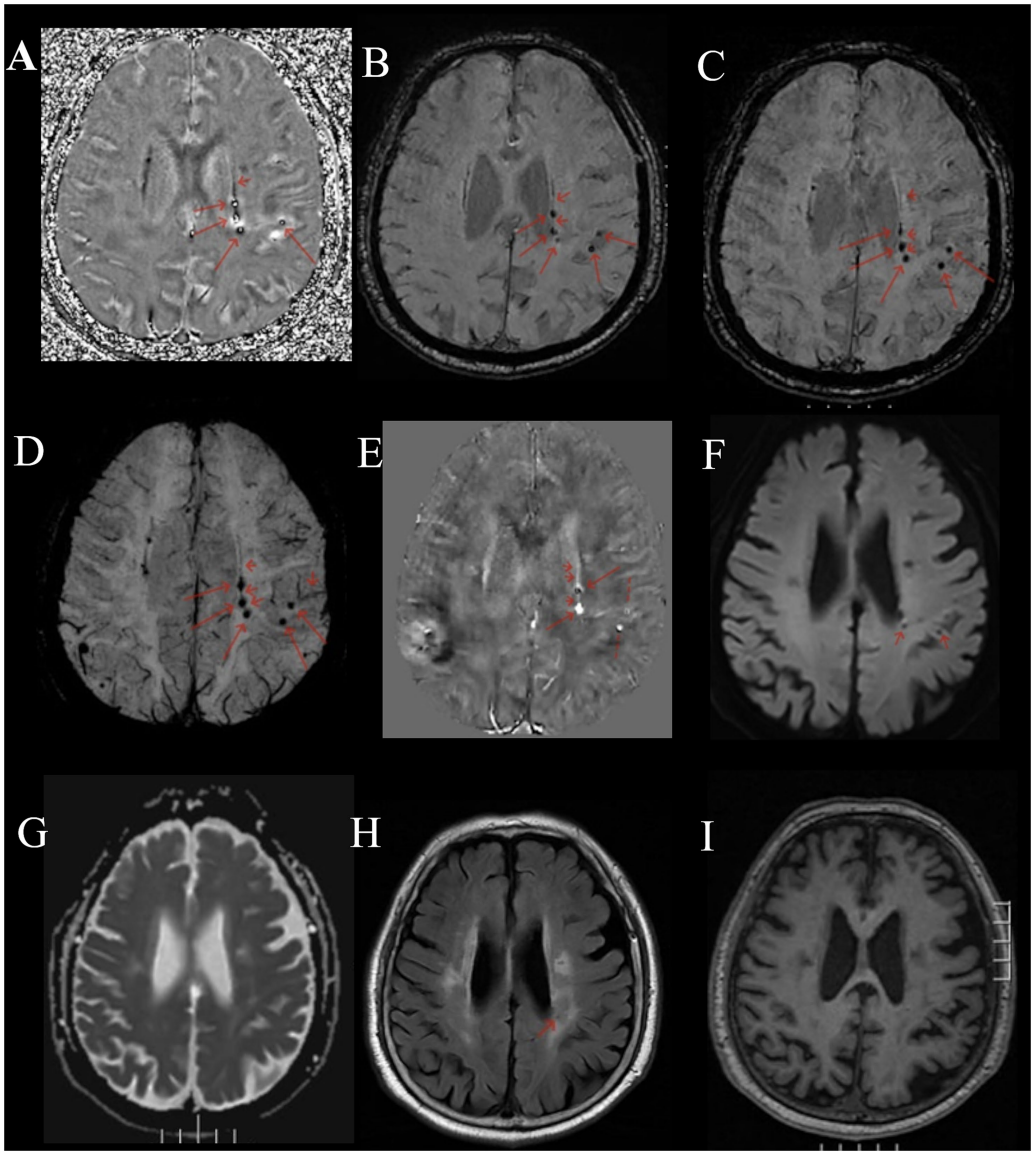
Male, 73 years old, with right limb motor impairment for 15 years and dizziness for 1 day. **(A)** Phase map. **(B)** Amplitude map. **(C)** SWI image. **(D)** MinIP image with TE of 22.5 ms. **(E)** QSM image. **(F)** DWI image. **(G)** ADC map. **(H)** FLAIR image. **(I)** T1WI image. **(A–D)** Round-like hypointense signals can be seen in the left periventricular region and the left parietal cortex, with hyperintense signals in the center, forming a typical “target sign” (long red arrow). The surrounding lesions show hypointense signals from the drainage of the left subependymal vein and parietal cortical vein (short red arrow). **(E)** QSM shows two round-like abnormal signals in the left periventricular region. The central part is the hyperintense central vein, the middle is hypointense, and the outermost edge is slightly hyperintense or hyperintense, forming the most typical “target sign” (long red arrow). The surrounding lesions have strip-like hyperintense subependymal vein drainage (short red arrow) passing through two BCTs (long red arrow). The left parietal cortex shows round-like lesions with hypointense in the center and hyperintense at the edge, forming a typical “target sign” (dashed long line). **(F)** DWI image, showing punctate hypointense signals in the left periventricular region and parietal cortex (short red arrow). **(G)** Shows slightly hyperintense signals. **(H)** Shows hypointense signals (short red arrow). **(I)** T1WI image, showing slightly hypointense signals.

**Figure 2:**
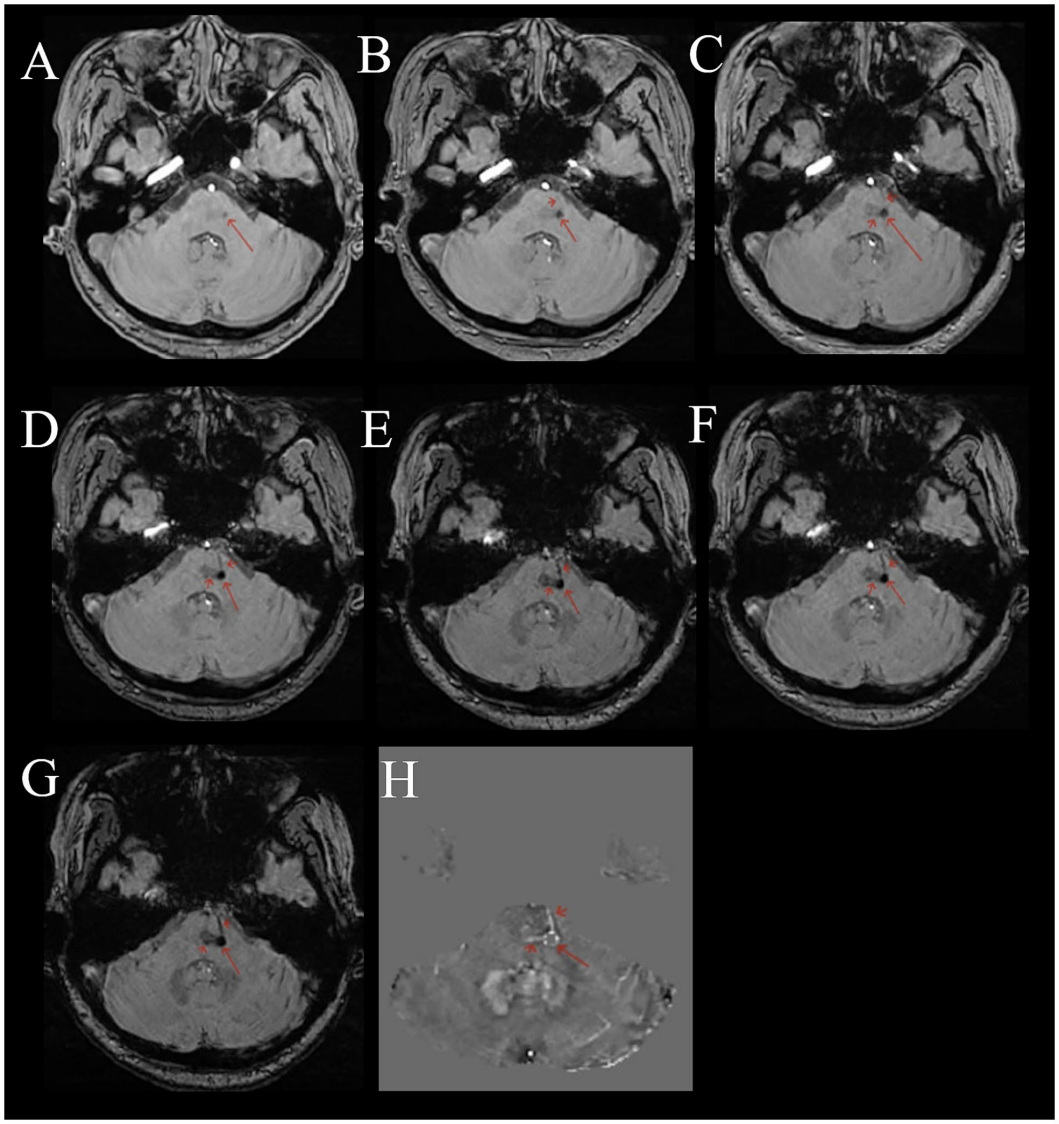
Female, 53 years old, with dizziness for 5 months. **(A–G)** Multi-echo QSM sequences with TE values of 3.3, 6.5, 9.7, 12.9, 16.1, 19.3, and 22.5 ms, respectively. **(H)** A QSM image. **(A–G)** Show a round low signal area in the pons (long red arrow). The surrounding area shows thickened, multiple draining veins with low signal (short red arrow). As the TE time increases, the lesion and draining veins become clearer. **(H)** Shows a disordered round signal in the pons (long red arrow), with surrounding draining veins showing high signal (short red arrow).

Compared to normal or other disease (see Supplementary material), on multi-echo SWI with long TE (22.5 ms), BCT lesions all presented as distinct small round hypointense signals on images, with punctate or strip-like slightly hyperintense signals at the center and a hypointense ring at the edge, forming a typical “single-ring target sign” (long red arrows in Figures 1A–D, 2A–G). Lesions had clear boundaries, and increased and thickened draining small veins were visible around the lesions (short red arrows in Figures 1A–D, 2A–G). QSM images showed round abnormal signals of lesions, with the central part being hyperintense due to the passage or penetration of central veins, a hypointense ring in the middle, and slightly hyperintense or hyperintense signals at the outermost edge, forming the most typical “double-ring target sign” (long red arrow in Figure 1E). The edges of some lesions showed a slightly hyperintense ring, and the inner side showed a hypointense ring, forming a typical “target sign” (dashed red arrow in Figure 1E). Meanwhile, increased and thickened small veins were observed around the lesions (short red arrows in Figures 1E, 2H). Some lesions only showed punctate hyperintense signals (solid long red arrow in Figure 1E). DWI, Flair, and T1WI images showed a few punctate hypointense signals (short red arrows in Figures 1F,H,I), and ADC showed punctate hyperintense signals (Figure 1G).

As shown in Supplementary Table S2, the inter-reader agreement was almost perfect agreement for SWI ( κ>0.8 ) and QSM ( κ>0.8 , except for cerebellum κ=0.741 ), whereas DWI demonstrated substantial agreement ( κ>0.6 ). In 36 cases of BCT, the number of lesions ranged from 1 to 109, including 6 cases with 1 lesion, 2 cases with 2 lesions, and 28 cases with multiple lesions. Among them, 23 cases (63.8%) were in the brain stem, 22 cases (61.1%) in the thalamus, 20 cases (55.6%) in the basal ganglia, 7 cases (19.4%) in the frontal lobe, 6 cases (16.7%) in the parietal lobe, 15 cases (41.7%) in the temporal lobe, 14 cases (38.9%) in the occipital lobe, and 15 cases (41.7%) in the cerebellar hemisphere. The QSM sequence with long TE (22.5 ms) had the highest efficiency in detecting BCT, followed by the QSM map, and the DWI image detected fewer lesions. Taking the multi-echo SWI sequence (TE 22.5 ms) as the gold standard for detecting BCT, the positive rate of DWI for BCT was 36.9%, that of the QSM map was 85%, and the positive rate of QSM was better than that of DWI, with a statistically significant difference ( $\chi^{2}$ = 334.609, *p* < 0.001). The lesions were mainly distributed in the brain stem, thalamus, and basal ganglia, and could also occur in various parts of the brain (Table 1).

**Table 1:** Comparison of the detection rate and sensitivity of BCT in 36 cases using multiple MR sequences, along with the distribution and number of BCTs in various regions.

Sequence	Thalamus	Basal ganglia	Brainstem	Frontal lobe	Parietal lobe	Temporal lobe	Occipital lobe	Cerebellum	Total	The positive rate
DWI	93	59	43	2	3	12	13	29	254	36.9%
SWI (TE22.5 ms)	167	132	99	21	57	64	78	70	688	100%
QSM	155	113	70	19	35	51	74	68	585	85.0%

### Comparison of the sensitivity of SWI and QSM map in detecting the central vein of BCT

The multi-echo SWI with long TE (22.5 ms) showed that among the 688 detected BCT lesions, 498 could display the central vein, with a positive rate of 72.4%. The QSM map detected 585 BCT lesions, among which 324 could display the central vein, with a positive rate of 55.4%. There was a difference in the detection rate of the central vein of BCT between the two sequences, and the multi-echo SWI sequence (TE 22.5 ms) was better than the QSM map ( $\chi^{2}$ = 39.937), (*p* < 0.001)) (Figure 2) (see Table 2).

**Table 2:** Comparison of the detection rate of BCT and the sensitivity of central veins in 36 cases using multi-echo SWI sequences with long TE (22.5 ms) and QSM image.

Sequence	No. of lesions	No. of central vein
SWI	688	498
QSM	585	324

## Discussion

In 1941, Blackwood first described two histologically confirmed cases of BCT (21). Histologically, this malformation consists of a cluster of dilated, tortuous thin-walled capillaries surrounded and separated by normal brain tissue. No gliosis, calcification, or hemosiderin deposition was observed. BCT predominantly occurs in middle-aged and elderly individuals, with an average age of 64.75 ± 11.08 years in this group. Literature reports that BCT is rare (2, 22, 23), most commonly occurring in the pons (24–27), although some reports indicate it is most common in the posterior fossa (28). With the widespread clinical application of SWI and QSM, a search of our hospital’s records from January 1, 2023, to July 31, 2024, revealed 1,705 cases of SWI or QSM examinations, among which 385 cases were BCT, accounting for 22.58%. On average, there were 0.67 cases per day. Among the 36 BCT patients examined with multi-echo SWI technology, 28 had multiple lesions. The most common lesion sites were the brainstem, thalamus, and basal ganglia (63.8, 61.1, and 55.6%, respectively).

Susceptibility-weighted imaging (SWI) technology was first invented by Haacke et al. (29) in 1997 and patented in 2002. Initially termed “high-resolution blood oxygen level-dependent venography,” was renamed “susceptibility-weighted imaging” in 2004, as it enables the reflection of more comprehensive information on material magnetic susceptibility properties. Early applications of this technology primarily focused on displaying small intracranial veins. Literature reports confirmed through follow-up that MRI findings in BCT patients showed that due to the occult nature of BCT and low blood flow in the vessels, the flow void effect of the vessels was not obvious. Conventional MRI could not display the flow void vessels, with most lesions appearing isointense on T1WI and T2WI, slightly hyperintense on FLAIR, and showing multiple punctate hypointensities on DWI, with a detection rate of only 36.9% (22, 30, 31). This led to high rates of misdiagnosis and missed diagnosis. BCT is also difficult to detect in conventional imaging. SWI special sequences began to be applied clinically in 2004 and were not routine examinations, and the understanding of BCT lesions was limited. For these reasons, the true prevalence of BCT remains unknown. Literature is relatively outdated or new literature references old reports indicating that BCT is rare (2, 22, 23, 28–33). With the widespread use of high-resolution 3.0 T MRI and 32- and 64-channel head phased-array surface coils, the detection rate of BCT has increased. In our hospital, 385 cases of BCT were diagnosed in the past 19 months, averaging 0.67 cases per day, indicating that BCT is not a rare entity but a relatively common benign vascular variant.

In recent years, the application of high-resolution 3.0 T MRI and continuous improvements in related technologies have greatly expanded its clinical application range. United Imaging in China developed the multi-echo susceptibility-weighted imaging sequence (swiplus) technology, which is based on a three-dimensional gradient multi-echo fusion sequence (20, 34–39). By using multiple (7) TEs and shortening TR, it achieves more significant T2* contrast, leading to local magnetic field inhomogeneity and increased magnetic susceptibility differences between tissues. The multi-echo gradient echo sequence fully utilizes the positive effects of long TE on magnetic susceptibility, reducing almost all distortions of single-echo long TE scans and outperforming traditional single-echo SWI (36, 40). The multi-echo gradient echo sequence shows higher contrast-to-noise ratio and better image quality, making small cerebral veins more prominent (40). Additionally, swiplus has flow-sensitive black blood (FSBB) imaging with diffusion gradients, highlighting magnetic susceptibility differences between tissues (32, 33). The QSM derived from the swiplus reflects the phase information allowing effective lesion detection (41–43).

A healthy vascular network has high blood flow arteries (containing oxyhemoglobin) between capillary clusters, and low blood flow venous capillaries connect arteries and veins, serving as the main site for material exchange between blood and surrounding tissue cells (containing deoxyhemoglobin) (44). Oxyhemoglobin is diamagnetic, while deoxyhemoglobin is paramagnetic. BCT consists of a cluster of dilated, tortuous thin-walled capillaries with low blood flow venous blood and scattered normal brain tissue. As the dilated, tortuous, and congested capillaries worsen, the concentration of deoxyhemoglobin in the venous blood increases, leading to greater magnetic field gradients and magnetic susceptibility (45, 46). This causes increased local magnetic field distortion and a significant magnetic susceptibility blooming effect. The multi-echo SWI sequence (TEs of 3.3, 6.5, 9.7, 12.9, 16.1, 19.3, and 22.5 ms) selects short TEs to display intracranial arteries, with oxyhemoglobin in the arteries showing high signal on QSM sequences. Longer TEs improve SNR in field maps and increase sensitivity to magnetic susceptibility. As TE time increases from short to long, slow-flowing small vessels and dilated capillaries gradually become clear, consistent with literature reports (41, 43, 47). Capillaries and veins show signal on QSM sequences, appearing as high signal on QSM maps. The QSM sequence has a slice thickness of 0.8 mm with no gap, while conventional MRI and traditional 2D GRE have a slice thickness of 5 mm with a 1.5 mm gap. Since BCT lesions are usually small, thicker slices often affect lesion detection. The external magnetic field strength and the magnetization intensity of tissues entering the external magnetic field determine tissue magnetic susceptibility. Therefore, with a fixed tissue magnetization intensity, the external magnetic field strength is the key factor determining magnetic susceptibility. SWI contrast is field strength-dependent, so high-resolution 3.0 T MRI with 32- and 64-channel head phased-array surface coils provides superior SWI image contrast compared to 1.5 T and 4- or 8-channel head phased-array surface coils (1, 48).

In this group, 36 BCT patients underwent multi-echo SWI examinations. The multi-echo SWI sequence TEs were 3.3, 6.5, 9.7, 12.9, 16.1, 19.3, and 22.5 ms. At long TE (22.5 ms), BCT patients showed clear single or multiple punctate, small round, or ring-shaped hypointensities in the brain parenchyma with clear edges. Most lesions appeared as round or ring-shaped hypointensities with slightly hyperintense centers, forming the typical “single ring target sign,” indicating central veins passing through dilated capillaries. Increased and thickened draining capillaries or capillaries passing through the lesions were visible around the lesions, with varying sizes and some lesions merging. QSM maps showed round abnormal signals, with the central high signal representing central veins, the intermediate low signal possibly indicating the conversion process from oxyhemoglobin to deoxyhemoglobin or lower deoxyhemoglobin content, and the outer slightly high or high signal indicating increasing deoxyhemoglobin, forming the typical “double ring target sign.” Some lesions appeared round with central low signal and peripheral high signal, forming the typical “single ring target sign.” Small lesions showed punctate high signal without target signs, easily diagnosed as microhemorrhages, but central veins were visible on long TE echoes. In this group, long TE (22.5 ms) SWI showed that the multi-echo SWI sequence (long TE 22.5 ms) was superior to QSM maps (*χ*^2^ = 39.937, *p* < 0.001). The QSM sequence only showed deoxyhemoglobin, but QSM maps could display the material exchange between blood and surrounding tissue cells in BCT lesions, indicating the conversion process from oxyhemoglobin to deoxyhemoglobin and the deoxyhemoglobin content.

BCT lesions on SWI show the typical “single ring target sign,” while QSM maps show the typical “double ring target sign” or “single ring target sign” with increased and thickened draining veins around the lesions. Lesions are small and visible on multiple consecutive slices, with some lesions merging. CMB and BCT have similar signal characteristics, often leading to misdiagnosis of BCT as CMB. CMB appears as punctate hypointensities on SWI and punctate hyperintensities on QSM maps, without the typical “single ring target sign” or “double ring target sign,” and lesions are visible on only 1–2 slices.

In summary, the multi-echo SWI sequence highlights the susceptibility differences between tissues and improves spatial resolution, thus effectively detecting lesions by increasing the longer TE and shortening the TR, which is the most sensitive sequence for diagnosing BCT and lesion visualization, has the potential to serve as the gold standard for BCT diagnosis.

## Data Availability

The raw data supporting the conclusions of this article will be made available by the authors, without undue reservation.
