# Supplementary material for: Application of multi-echo susceptibility weighted imaging in the evaluation of brain capillary telangiectasias
Source: Front Neurol. 2025 Nov 3;16:1593152. doi: 10.3389/fneur.2025.1593152 (PMC12620245; doi:10.3389/fneur.2025.1593152)
Supplement: Supplementary file 1 [file Table_1.DOCX]

S1. The first image shows a normal QSM map where the anterior vein of corpus callosum, transverse veins, internal cerebral veins, and cortical veins present as hyperintense signals. The second image demonstrates a microbleed in the left thalamus. The third image shows a case of CAA (Cerebral Amyloid Angiopathy) with hemorrhage in the right parietal lobe and microbleeds in the left parietal lobe.


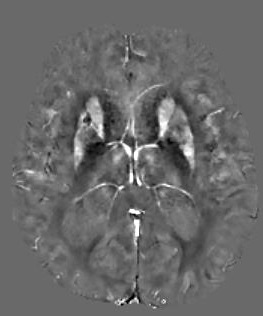

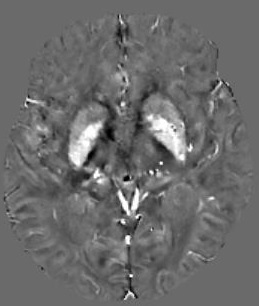

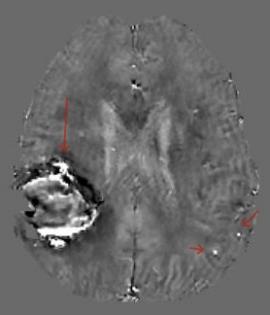


S2. Fleiss’s Kappa values for inter-reader agreement regarding DWI, SWI, and QSM at 8 anatomical locations.

|  | Thalamus | Basal ganglia | Brainstem | Frontal lobe | Parietal lobe | Temporal lobe | Occipital lobe | Cerebellum |
| --- | --- | --- | --- | --- | --- | --- | --- | --- |
| SWI | 0.958 | 0.917 | 0.952 | 0.893 | 0.878 | 0.870 | 0.948 | 0.914 |
| QSM | 0.893 | 0.874 | 0.880 | 0.920 | 0.889 | 0.891 | 0.949 | 0.741 |
| DWI | 0.657 | 0.641 | 0.659 | 0.811 | 0.767 | 0.598 | 0.866 | 0.708 |
